# Depletion of the heaviest stable N isotope is associated with NH_4_^+^/NH_3 _toxicity in NH_4_^+^-fed plants

**DOI:** 10.1186/1471-2229-11-83

**Published:** 2011-05-16

**Authors:** Idoia Ariz, Cristina Cruz, Jose F Moran, María B González-Moro, Carmen García-Olaverri, Carmen González-Murua, Maria A Martins-Loução, Pedro M Aparicio-Tejo

**Affiliations:** 1 Instituto de Agrobiotecnología, IdAB – CSIC - Universidad Pública de Navarra - Gobierno de Navarra, Campus de Arrosadía s/n, E-31006 Pamplona, Navarra, Spain; 2 Universidade de Lisboa, Faculdade de Ciências, Centro de Biologia Ambiental - CBA, Campo Grande, Bloco C-4, Piso 1, 1749-016 Lisboa, Portugal; 3 Department of Plant Biology and Ecology, Faculty of Science and Technology, University of Basque Country (UPV-EHU), Apdo. 644; E-48080 Bilbao, Vizcaya, Spain; 4 Department of Statistics and Operations Research, Public University of Navarre, Campus de Arrosadía s/n, E-31006 Pamplona, Navarra, Spain

**Keywords:** Low affinity ammonium transporters, Nitrogen isotopic signature, Ammonium/ammonia, Ammonium dissociation isotope factor, ammonia uptake

## Abstract

**Background:**

In plants, nitrate (NO_3_^-^) nutrition gives rise to a natural N isotopic signature (δ^15^N), which correlates with the δ^15^N of the N source. However, little is known about the relationship between the δ^15^N of the N source and the ^14^N/^15^N fractionation in plants under ammonium (NH_4_^+^) nutrition. When NH_4_^+ ^is the major N source, the two forms, NH_4_^+ ^and NH_3_, are present in the nutrient solution. There is a 1.025 thermodynamic isotope effect between NH_3 _(g) and NH_4_^+ ^(aq) which drives to a different δ^15^N. Nine plant species with different NH_4_^+^-sensitivities were cultured hydroponically with NO_3_^- ^or NH_4_^+ ^as the sole N sources, and plant growth and δ^15^N were determined. Short-term NH_4_^+^/NH_3 _uptake experiments at pH 6.0 and 9.0 (which favours NH_3 _form) were carried out in order to support and substantiate our hypothesis. N source fractionation throughout the whole plant was interpreted on the basis of the relative transport of NH_4_^+ ^and NH_3_.

**Results:**

Several NO_3_^-^-fed plants were consistently enriched in ^15^N, whereas plants under NH_4_^+ ^nutrition were depleted of ^15^N. It was shown that more sensitive plants to NH_4_^+ ^toxicity were the most depleted in ^15^N. In parallel, N-deficient pea and spinach plants fed with ^15^NH_4_^+ ^showed an increased level of NH_3 _uptake at alkaline pH that was related to the ^15^N depletion of the plant. Tolerant to NH_4_^+ ^pea plants or sensitive spinach plants showed similar trend on ^15^N depletion while slight differences in the time kinetics were observed during the initial stages. The use of RbNO_3 _as control discarded that the differences observed arise from pH detrimental effects.

**Conclusions:**

This article proposes that the negative values of δ^15^N in NH_4_^+^-fed plants are originated from NH_3 _uptake by plants. Moreover, this depletion of the heavier N isotope is proportional to the NH_4_^+^/NH_3 _toxicity in plants species. Therefore, we hypothesise that the low affinity transport system for NH_4_^+ ^may have two components: one that transports N in the molecular form and is associated with fractionation and another that transports N in the ionic form and is not associated with fractionation.

## Background

Nitrogen (N) and carbon (C) are the main components of all living organisms and regulate the productivity of most ecosystems. In agriculture, N is by far the main nutrient in fertilisers, with nitrate (NO_3_^-^) and ammonium (NH_4_^+^) being the main N sources used by plants. However, relatively little is known about the isotopic fractionation during uptake of these ions. Assessment under natural conditions is difficult because, under most circumstances, NO_3_^- ^and NH_4_^+ ^are simultaneously present in the soil and their concentrations change both spatially and temporally over a wide range (e.g., 20 μM to 20 mM) [1,2]. Furthermore, this situation becomes even more complex if the rhizosphere and its symbiotic interactions (N_2_-fixing organisms or mycorrhiza) are taken into account.

The natural variation in stable N isotopes has been shown to be a powerful tool in several studies of plant and ecosystem N dynamics [3]. Generally, the global δ^15^N value of the plant biomass is determined by that of the primary N source (soil N, fertiliser, N_2_) [4]. Some studies assume that the δ^15^N of leaf tissue reflects that of the source in the soil (e.g., see [5]). This assumption implies that the isotope ratio of the N source is preserved during N absorption, assimilation and translocation. However, it is clear that physiological processes and biological mechanisms, such as N-uptake, assimilation through distinct pathways, internal N recycling in the plant and gaseous N exchange, can discriminate against ^15^N [4]. Furthermore, plant N fractionation is also dependent on the N availability. Thus, in the case of unlimited substrate (N) availability, an isotope effect will always be expressed, and therefore, the arising δ^15^N will be lower than in the N source if fractionation occurs [6]. In contrast, in a growth system where the quantity of substrate (N) is limited, and the organism exhausts the N source completely, the plant δ^15^N will be similar (or even identical) to the original N source [6,7]. Most studies concerning physiological and natural N fractionation have involved plants grown with NO_3_^- ^as the only N source. A review of these studies [6] showed that N fractionation changes with plant age, the external NO_3_^- ^concentration and the partitioning of N metabolism between the roots and shoots.

Similarly to NO_3_^-^, NH_4_^+ ^influx through the membrane of plant cells exhibits a predominantly biphasic pattern. Thus, at concentrations up to 0.5-1 mM N, influx occurs *via *the high affinity transport system (HATS), which is saturable and energy dependent and has a *K_m_* in the submillimolar concentration range; the non-saturable low affinity transport system (LATS) operates with a *K_m_* in the millimolar concentration range, i.e., at N concentrations above 0.5-1 mM, for most plant roots [8,9].

While the proteins responsible for the high-affinity NH_4_^+ ^transporters have been identified in many plant species, the low-affinity uptake system proteins have yet to be identified [9]. Recently, Loqué and von Wirén reviewed the different levels at which NH_4_^+ ^transport is regulated in plant roots under HATS conditions [10]. A functional analysis of several ammonium transporters (AMTs) expressed in *Xenopus *oocytes showed evidence that NH_4_^+^, rather than NH_3_, uniport is the most likely transport mechanism for AMT1-type transporters from plants [11-13]. Nevertheless, individual plant AMT/Rh transporters may use different transport mechanisms [13] compared with the AMT2-type transporters, which recruit NH_4_^+^-mediated electroneutral NH_4_^+ ^transport, probably in the form of NH_3_[14,15].

On the contrary, the molecular basis of transport under LATS conditions remains poorly understood. LATS for NH_4_^+ ^operates when NH_4_^+ ^is present at high concentrations in solution; under these conditions, several symptoms of toxicity have often been observed in a broad range of plant species [2]. Few studies have examined the natural isotopic signature of plants grown with NH_4_^+ ^nutrition under LATS conditions and its relationship with sensitivity or tolerance to NH_4_^+ ^nutrition. It has been speculated that NH_3 _could be the chemical species that enters the plant from the external medium *via *the plasma membrane [7,16]. Under conditions of high external pH and high NH_4_^+^, the transport of NH_3 _across membranes occurs, and it can become biologically significant [16,17]. In agro-ecosystems, in which the soils are currently fertilised with urea (50% of the total world fertiliser N consumption [18]) or (NH_4_)_2_SO_4_, emissions of N in the NH_3 _form take place (i.e., up to 10-20% of N in fertilisers applied as urea may be lost in the soil [19]). Thus, under these conditions, significant amounts of NH_3 _may be present in the soil and therefore enter the plant. When NH_4_^+ ^is applied as the only N source or NH_4_^+ ^is formed naturally in soils *via *mineralization of organic matter, the two forms, NH_4_^+ ^and NH_3_, are present in the nutrient solution. The neutral and ionic forms do not have exactly the same natural isotopic signatures because there is a 1.025 thermodynamic isotope effect between NH_3 _(g) and NH_4_^+ ^(aq), so NH_3 _(aq) is depleted for ^15^N by 20‰ relative to NH_4_^+ ^(aq) [20]; in addition, the equilibrium fractionation factor for exchange of NH_3 _(aq) with NH_3 _(g) has been estimated as ~ 1.005 [21].

Thus, an understanding of the physiological processes that lead to variations in the stable isotopic composition is required. This work was intended to assess the natural δ^15^N dynamics for several plant species grown hydroponically under controlled conditions and with only one N source, namely NO_3_^- ^or NH_4_^+^. Our working hypothesis for this study was that a part of NH_4_^+ ^enters the plant root as neutral molecules (i.e. NH_3_) favouring the isotopic fractionation and this fractionation process during NH_4_^+ ^uptake is related to the sensitivity of plants to NH_4_^+ ^nutrition. Fractionation of the N source throughout the whole plant was interpreted on the basis of the relative transport of NH_4_^+ ^and NH_3_. We also propose that LATS for NH_4_^+ ^uptake may have two components, one that involves the ionic form (NH_4_^+^) and another that involves the molecular form (NH_3_).

## Methods

### Plant Culture

#### i) Isotopic signature experiment in several plant species

Nine species that show different NH_4_^+ ^tolerances were grown hydroponically with NH_4_^+ ^or NO_3_^- ^as the sole N sources. Lettuce (*Lactuca sativa *L. cv. Marine), spinach (*Spinacia oleracea *L. cv. Spinner), tomato (*Solanum lycopersicum *L. cv. Trust), pea (*Pisum sativum *L. cv. Eclipse) and lupin (*Lupinus albus *L. cv. albus) plants were germinated, cultured and treated as described previously [22]. Carob (*Ceratonia siliqua *sp.) and *Acacia aneura *sp. plants were grown according to [23]. Perennial ryegrass (*Lolium perenne *L. cv. Herbus) and white clover (*Trifolium repens *L. cv. Huia) were cultured according to [24]. Pea plants (cv. Sugar-snap) were grown according to [25], and spinach (cv. Gigante de invierno) and pea plants (cv. Rondo) were cultured as described in [24]. Plants from each species were divided into two groups, each of which received different concentrations of N (0.5 to 6.0 mM) in the form of either NO_3_^- ^or NH_4_^+ ^(applied as Ca(NO_3_)_2 _or KNO_3 _and (NH_4_)_2_SO_4_, respectively). All seeds were surface-sterilised and plants were grown for several days (depending on the plant species) under hydroponic conditions. The pH of the nutrient solutions was buffered with CaCO_3 _(5 mM) to pH 6-7, depending on the plant species. The temperature of the solutions was between 18 and 20°C. Nutrient solutions were aerated vigorously (flow rate of 15 mL s^-1^) and replaced weekly to minimize the nitrification processes.

Plants were harvested by separating the shoots and roots of each plant. The dry weight of each plant was obtained after drying in an oven at 75-80°C to a constant weight (48-72 h).

#### ii) Short-term control and ^15^N labelling experiments in spinach and pea plants

Spinach seeds (cv. Gigante de Invierno) were germinated and grown hydroponically as described by [26]. N-free Rigaud and Puppo solution [27], which had been diluted (1:2) and modified according to [25] was used during the growth period. The N-free solution was supplemented with 0.5 mM NH_4_NO_3 _as the only N source for the first 25 days of growth period. Then, spinach plants were fed with a Rigaud and Puppo solution containing 0.5 mM NH_4_Cl as the only N source for the last 5 days of the growth period. The pH of the solution was buffered with CaCO_3 _(0.25 mM) to pH 6-6.5.

Pea seeds (cv. Sugar-snap) were surface-sterilised according to [28] and then germinated as described in [25]. One-week-old pea seedlings were transferred into tanks (volume: 8 L) in groups of eight and grown in controlled-environment chambers at 275-300 μmol photons m^-2 ^s^-1^, 22/18°C (day/night), 60/70% relative humidity and a 14 h light/10 h dark photoperiod for 1-2 weeks, until the second node stage was reached. The hydroponic vessels contained aerated (0.4 L air min^−1 ^L^−1^) N-free Rigaud and Puppo solution [27], which had been diluted (1:2) and modified according to [25]. A solution of 0.5 mM NH_4_^+ ^was supplied as NH_4_Cl during the growth period as the only N source. The pH of the solution was buffered with CaCO_3 _(2.5 mM) to 7-7.3.

Either spinach or pea plants were then transferred to a solution at pH 6 (KP buffer, 10 mM) or pH 9 (H_3_BO_3_/NaOH buffer, 50 mM) in a sealed 125-ml Erlenmeyer flask, such that the roots were fully immersed in 100 mL of solution. Fully ^15^N-labelled ^15^NH_4_Cl was injected and rapidly mixed to a final concentration of 10 mM NH_4_^+^. Plants from both pH levels were harvested by separating the shoots and roots of each plant at 0, 1, 7.5 (for spinach), 15, 30, 60 and 120 min after the ^15^NH_4_Cl injection. In order to evaluate how the pH increase affects ion uptake *per se*, we have used as control a nutrient solution containing RbNO_3 _(1 mM), instead of ^15^NH_4_Cl. This control was performed exclusively on spinach, which is considered a more sensitive species than pea. Internal Rb^+ ^and NO_3_^- ^contents were determined in shoots and roots at 7.5, 30 and 120 min after RbNO_3 _injection, as tracers of cation and anion uptake respectively in different pHs.

For the uptake experiments, the applied light intensity during the pH and RbNO_3 _or ^15^N-labelling short-term applications was 750-800 μmol photons m^-2 ^s^-1 ^to enhance the absorption process.

pH measurements were determined after the short-term experiments in order to verify that the pH of the solution was properly buffered and that there were no great changes in the pH due to the root ionic exchanges (ion influx/efflux) (Additional file 1).

### Isotopic N Composition and N accumulation

Five to eight milligrams of powdered plant material from each sample (shoots and roots) was separately packed in tin capsules. The ^15^N/^14^N isotope ratios of these samples were determined by isotope ratio mass spectrometry (isoprime isotope ratio mass spectrometer - IRMS, Micromass-GV Instruments, UK). The N isotope composition results are expressed as δ^15^N, in parts per thousand (‰) relative to atmospheric N_2_: δ^15^N (‰) = [(R_sample_/R_standard_)-1] * 1000, where R_sample _is the ^15^N/^14^N ratio of the sample and R_standard _is the ^15^N/^14^N ratio of the atmospheric N_2_. Plant material that had previously been calibrated against a standard material of known isotope composition was used as a working standard for batch calibration during the isotope ratio analyses. The ^15^N contents (total, ^15^NH_4_^+ ^and ^15^NH_3_) were obtained using δ^15^N and the total percentage of N for each plant tissue (leaves and roots), and ^15^N contents for the external NH_4_^+ ^and NH_3 _were calculated using the Henderson-Hasselbalch equation, which takes into account the external pH. The percentages of NH_3 _molecules (relative to the total [NH_4_^+ ^+ NH_3_] molecules) at pH 6.08 and pH 9.0 were 0.0676% and 35.993%, respectively (see Additional file 2). Plant tolerance to NH_4_^+ ^nutrition was calculated as the ratio between biomass accumulation of NH_4_^+^- and NO_3_^-^-fed plants at the same N concentration [22]. The δ^15^N data corresponding to the N sources used ranged from +0.03 to +2.31 for NH_4_^+ ^and -1.514 to +0.3 ‰ for NO_3_^-^.

### Determination of inorganic soluble ion content

Plant extracts with soluble ionic contents from shoots and roots were obtained from dry tissues incubated in a bath in 1-2 mL of milli-Q water at 85°C for 10 min, followed by centrifugation (20,000×*g*, 30 min). The supernatants were stored at -20°C until analysis by ion chromatography. Soluble cation content (Rb^+^) was determined as described in [27] using an isocratic method with 20 mM metanosulphonic acid solution. Soluble anion content (NO_3_^-^) determination was carried out by the gradient method given by [27]. Rb^+ ^content was below the detection limit in shoots.

### Statistical analyses

All statistical analyses were performed with Statistical Product and Service Solutions (SPSS) for Windows, version 17.0.

#### i) Statistical analysis of the natural isotopic abundance experiment in several plant species

We examined results for nine species using analysis of variance to test for effects and interactions of the N treatments (source and concentration) and whether these changed according to the organ and species tested. Organ was included as a factor exclusively in the natural isotopic composition ANOVA test because it was meaningless to include it in the total biomass and total biomass ratio (NH_4_^+^/NO_3_^-^) ANOVA tests.

#### ii) Statistical analysis for short-term experiments in spinach and pea plants

One-way analysis of variance (ANOVA; factor: time) was performed. The homogeneity of variance was tested using the Levene test [29]. Least significant difference (LSD) statistics were applied for variables with homogeneity of variance, and the Dunnett T3 test [30] was used for cases of non-homoscedasticity. The pHs were compared using Student's *t*-test for each time point independently, and homoscedasticity was determined using the Levene test [29].

All statistical analyses were conducted at a significance level of 5% (*P *≤ 0.05). The results of this study were obtained for plants cultured in several independent series. For the plant species lettuce (cv. Marine), spinach (cv. Spinner), tomato (cv. Trust), pea (cv. Eclipse) and lupin (cv. Albus), plant material from six plants was mixed and analysed in three independent series. For spinach (cv. Gigante de invierno), pea (cv. Sugar-snap and Rondo), carob, perennial ryegrass (cv. Herbus), white clover (cv. Huia) and *Acacia *sp., at least one sample was analysed for each of three independent series.

## Results

**Figure 1:**
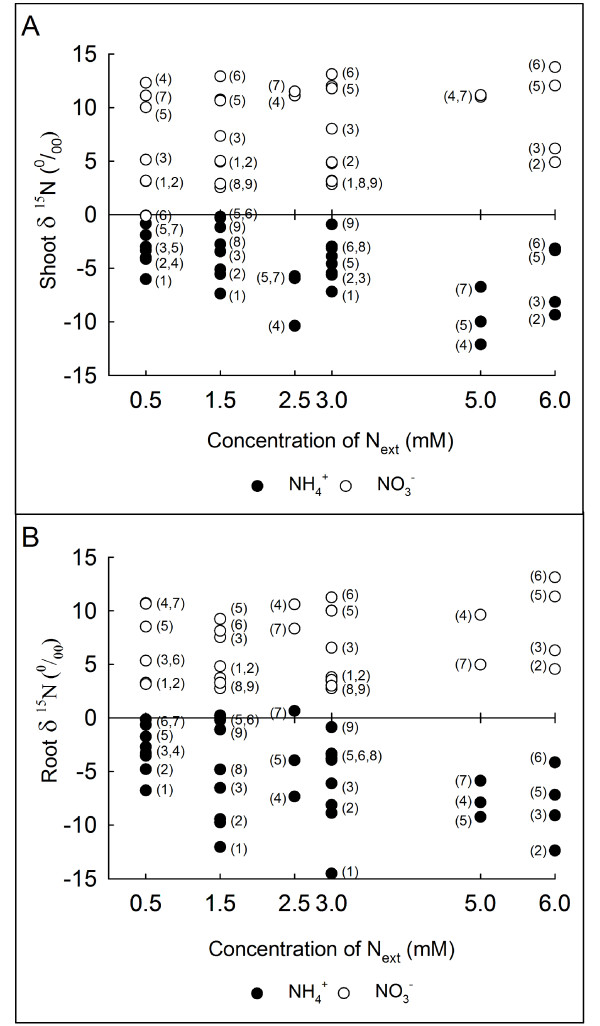
**Natural N isotopic composition of nine plant species with different sensitivity to NH_4_^+ ^nutrition**. Natural isotopic signatures (δ^15^N, ‰) of the shoots **(A) **and roots **(B) **of several plant species cultured under hydroponic conditions with different concentrations of NH_4_^+ ^(●) or NO_3_^- ^(○) as the sole N source. The following numbers indicate the species that correspond to each point: (1) *Lactuca sativa *L., (2) *Spinacia oleracea *L., (3) *Solanum lycopersicum *L., (4) *Lolium perenne *L., (5) *Pisum sativum *L., (6) *Lupinus albus *L., (7) *Trifolium repens *L., (8) *Ceratonia siliqua *sp., and (9) *Acacia aneura *sp. Each point is the average of several biological replicates (at least n = 3, depending on the species; see Methods). δ^15^N of the N sources: NO_3_^- ^= +0.3 and -1.514 and NH_4_^+ ^= +0.029, +0.5 and +2.31 ‰.

Although the δ^15^N values of the sources, NO_3_^- ^and NH_4_^+^, similarly ranged from -1.514 to +2.31 ‰, the δ^15^N observed for several plant species was significantly different when N was provided either as NO_3_^- ^or NH_4_^+ ^(Table 1). In general, four trends emerged from the natural isotopic signature data (Figure 1): 1) NO_3_^-^-fed plants tended to be enriched in the heavier N isotope, whereas NH_4_^+^-fed plants were depleted compared with their respective N sources; 2) for the same external N concentration, the degree of fractionation depended on the plant species; 3) the δ^15^N values of shoots and roots were not the same but followed similar patterns; and 4) in contrast to the NO_3_^-^-fed plants, which had δ^15^N values that were insensitive to the N concentration, under NH_4_^+ ^nutrition, fractionation tended to increase with the N concentration within plant species (Table 2). These four trends were supported by the results displayed in Tables 1 and 2 from the analyses of variance of N, species and organ effects. The source of N had a global effect on the isotopic composition (‰) and total biomass (g DW) (Table 1). Moreover, significant two-way interactions between the N source and N concentration (N source × N conc.) and the N source and species (N source × sp.) on the δ^15^N and the total biomass were observed (Table 1). Due to the strong effect of the N source on the δ^15^N, the main effects of N concentration, species and organ type was analysed in NO_3_^-^- and NH_4_^+^- fed plants separately (Table 2). In NH_4_^+^-fed plants, the N concentration, species and organ type had an effect on the natural isotopic abundance; however, in NO_3_^-^- fed plants, only the diversity (species) factor had an effect on the δ^15^N (Table 2).

**Table 1:** Analysis of variance of the N sources, N concentrations and species.

*Global Effect*	δ ^15^N (‰)		Total Biomass (g DW)	
		
*Factor*	*F*	*P > F*	*F*	*P > F*
N Source	**1273.54**	**< 0.0001**	**8.62**	**0.0043**
N Source × N Conc.	**19.95**	**< 0.0001**	**16.01**	**< 0.0001**
N Source × sp.	**10.01**	**< 0.0001**	**39.71**	**< 0.0001**
N Source × N Conc. × sp.	1.23	0.2701	**7.46**	**< 0.0001**
		
Whole model R^2^	0.956		0.939	

Global effects of N sources and interaction terms, including the N source effects, on isotopic composition (‰) and total biomass (g DW). N Conc.: N concentration; sp.: species. The main effects of the N concentration and species are not included because the results of the ANOVA test were masked by the strong N source effect. They are shown separately by the N source in Table 2. Significant effects (*P *≤ 0.05) are shown in bold.

**Table 2:** Analysis of variance of the N concentrations, species and organ effects.

*Factor*	δ ^15^N (‰)		Total Biomass (g DW)		Total Biomass Ratio (NH_4_^+^/NO_3_^- ^)	
			
*Effect on NO_3_^-^-fed plants*	*F*	*P > F*	*F*	*P > F*	*F*	*P > F*
N Conc.	0.78	0.4743	**38.53**	**< 0.0001**	**10.92**	**< 0.0001**
sp.	**13.20**	**< 0.0001**	**80.73**	**< 0.0001**	**64.81**	**< 0.0001**
N Conc. × sp.	1.18	0.3655	**4.26**	**< 0.0001**	1.43	0.1912
Organ	1.80	0.1966	-	-	-	-
			
Whole model R^2^	0.884		0.942		0.927	

***Effect on NH_4_^+^-fed plants***	*F*	*P > F*	*F*	*P > F*	*F*	*P > F*

N Conc.	**34.69**	**< 0.0001**	1.57	0.2183	**8.93**	**0.0005**
sp.	**17.73**	**< 0.0001**	**80.56**	**< 0.0001**	**59.10**	**< 0.0001**
N Conc. × sp.	0.93	0.5418	**6.84**	**< 0.0001**	1.40	0.1999
Organ	**4.76**	**0.0392**	-	-	-	-
			
Whole model R^2^	0.916		0.936		0.908	

The effects of N concentration and species (sp.) and the corresponding interactions are shown separately by the N source on the isotopic composition (‰), total biomass (g DW) and total biomass ratio (NH_4_^+^/NO_3_^-^-fed plants). The organs did not influence the N concentration interaction (N Conc. × Organ; P > 0.8) or the species interaction (sp. × Organ; *P *> 0.05) or N Conc. × sp. interaction (N Conc. × Sp. × Organ; P > 0.8) with either N source. The interaction terms, including the organ effects, are therefore not shown above. Significant effects (*P *≤ 0.05) are shown in bold text.

Biomass accumulation in NH_4_^+^- and NO_3_^-^-fed plants at the same N concentration was dependent on the N concentration in the root medium and on the plant species concerned (Table 2). The degree of the effect of the N concentration on the total plant biomass (growth stimulation with NO_3_^- ^nutrition or growth inhibition with NH_4_^+ ^nutrition) depended on the species, as shown by the significant interaction of N conc. × sp. for both N sources (Table 2).

**Figure 2:**
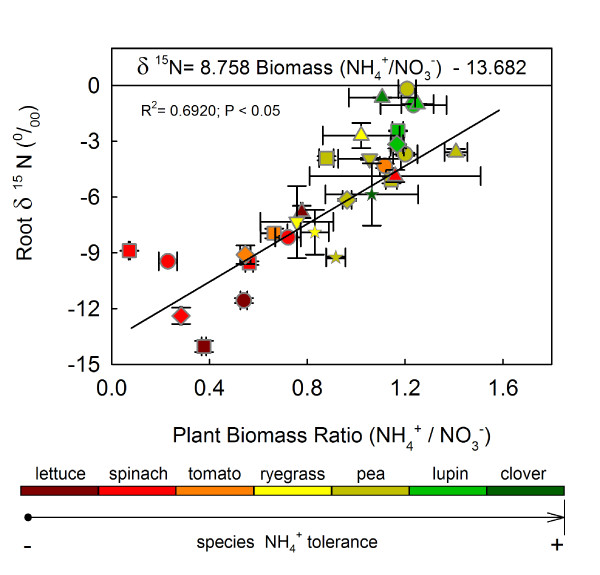
**Root isotopic signatures (δ^15^N, ‰) of NH_4_^+^-fed plants correlated with the plant NH_4_^+ ^toxicity/tolerance indicator (plant biomass ratio NH_4_^+^/NO_3_^- ^for each N concentration)**. The following N concentrations were represented in this analysis: 0.5 mM (upward triangle), 1.5 mM (circle), 2.5 mM (upside down triangle), 3 mM (square), 5 mM (star) and 6 mM (diamond). δ^15^N data of the (NH_4_)_2_SO_4 _used in NH_4_^+^-fed plants were +0.029, +0.5 and +2.31 ‰, and all three values fall within the area indicated (upper part of graph). The plant species that were cultured hydroponically and used for this statistical analysis were lettuce, spinach, tomato, ryegrass, pea, lupin and white clover. The dataset displayed represents the average values ± SE (at least n = 3, depending on species; see Methods). Linear regression was performed at *P *≤ 0.05.

The ratio of biomass accumulations between the NH_4_^+^- and NO_3_^-^-fed plants was therefore used as an indicator of each plant species' sensitivity (or tolerance) to NH_4_^+ ^nutrition. The N concentration and diversity also influenced the total biomass ratio of NH_4_^+^- and NO_3_^-^-fed plants (Table 2). A very strong correlation between the root δ^15^N of NH_4_^+^-fed plants and the ratio of biomass accumulation between the NH_4_^+^- and NO_3_^-^-fed plants was observed (Figure 2). Thus, the lower biomass ratios (i.e., lower tolerance to NH_4_^+^) observed for seven species and cultivars, which presented different degrees of tolerance to NH_4_^+ ^nutrition grown with several N concentrations, were associated with depletion of the heavier N isotope in the plant material studied (Figure 2). Hence, the most sensitive plants to NH_4_^+ ^were the most depleted of ^15^N (Additional file 3 table S1). The *Ceratonia *species (carob) showed a unique behaviour relative to the other herbaceous species; its much higher biomass ratios for the negative δ^15^N values did not fit within the correlation (see Additional file 3, table S1). The ratio of the whole plant biomass accumulation (NH_4_^+^/NO_3_^-^) in *Acacia *species was not measured. Hence, they were excluded from the dataset in Figure 2.

**Figure 3:**
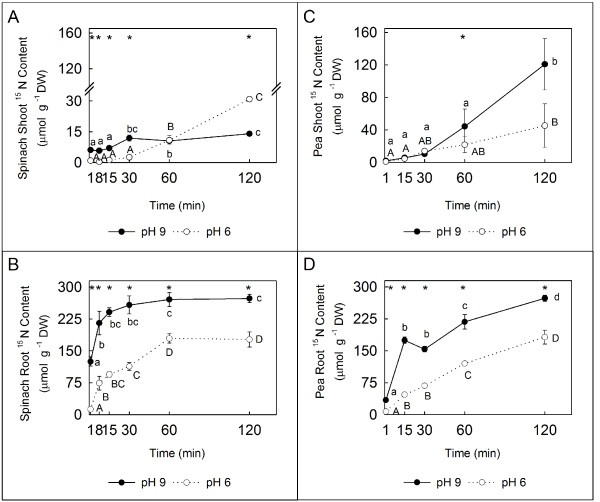
**^15^N contents in tissues of spinach and pea plants**. ^15^N content (μmol g^-1 ^DW) calculated from the δ^15^N data, in shoots (**A **and **C**) and roots (**B **and **D**) of spinach (**A **and **B**) and pea (**C **and **D**) plants transferred from pH 7 to pH 6 (○) or pH 9 (●).

**Figure 4:**
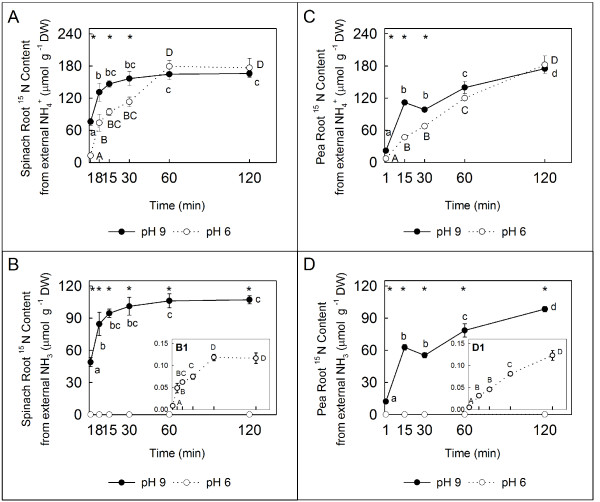
**Root ^15^NH_4_^+ ^and ^15^NH_3 _contents calculated from the total ^15^N uptake**. ^15^N content accumulated from ^15^NH_4_^+ ^absorption (μmol g^-1 ^DW) in spinach **(A) **and pea **(C) **plants. ^15^N content accumulated from ^15^NH_3 _absorption (μmol g^-1 ^DW) in spinach **(B) **and pea **(D) **plants. **(B1 **and **D1) **Magnified portions of plots (**B **and **D **respectively) showing the ^15^N content that accumulated as a result of external ^15^NH_3 _absorption at pH 6 (μmol g^-1 ^DW). The partitioning between NH_3 _and NH_4_^+ ^has been calculated using the Henderson-Hasselbalch equation (see Additional file 2). Data represent the average values ± SE (n = 3). Letters represent significant differences (*P *≤ 0.05) during exposure to pH 6 (A, B, C and D) and pH 9 (a, b, c and d). An asterisk (*) denotes significant differences between pH 6 and 9 (*P *≤ 0.05).

Natural soils rarely exhibit pH values close to the p*K*a of NH_4_^+ ^(~ 9.25); therefore, NH_3 _is present in very small amounts under normal external pH conditions [2]. In the short-term experiments described herein, three- and four-week-old N-deficient pea and spinach plants, respectively, were transferred to a 100% ^15^N-labelled 10 mM NH_4_^+ ^solution. δ^15^N was used as a tool to determine the amount of ^15^N that enters the plant roots under the experimental conditions, and a higher increase in the total ^15^N content was observed at pH 9 than at pH 6 in both plant species (Figure 3B and 3D). In plants with higher NH_4_^+ ^sensitivity, i.e., spinach, the ^15^NH_3_/^15^NH_4_^+ ^absorption reached the asymptotic trend moment in the curve in a shorter period of time than pea plants (Figure 3B and 3D). In shoots, the total ^15^N content per DW g was lower in spinach than in pea plants (Figure 3A and 3C). The content of ^15^N in spinach shoots was higher in pH 9 than in pH 6 (Figure 3A), whereas in pea plants no difference was observed between pHs during the initial 15 min (Figure 3C). This result indicates that in spinach plants the N is translocated immediately from the roots to the shoot, while in pea plants N translocation is delayed relative to N uptake. At 120 min, opposite effects between pHs were shown in both plant species. In spinach shoots, higher ^15^N content was displayed at pH 6, while pea shoots showed higher ^15^N content at pH 9 (Figure 3A and 3C). On the other hand, the internal root ^15^N content was related to the proportion of NH_4_^+ ^and NH_3 _in the external solution at pH 6 and 9 (Figure 4), as calculated using the Henderson-Hasselbalch equation (see Additional file 2). In both plant species, some important differences were found between the plants at pH 6 and 9 in terms of the proportion of ^15^N uptake from the external NH_4_^+ ^source during the initial 15 min after transfer to a different pH (Figure 4A and 4C), whereas the uptake rates of ^15^N from the external NH_4_^+ ^were similar at both pH levels 60 min after the beginning of the experiment (Figure 4A and 4C). The most remarkable finding, however, was a drastic increase in ^15^N uptake from the external NH_3 _source at pH 9, which was maintained throughout the experiment (up to 120 min, Figure 4B and 4D).

**Figure 5:**
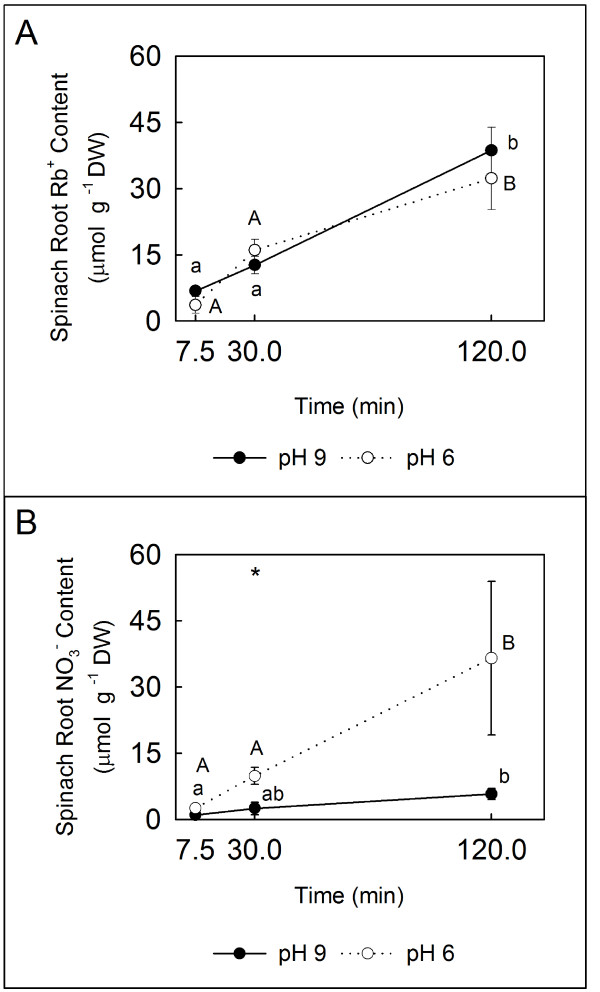
**Root ion contents of spinach plants**. Root ion content (μmol g^-1 ^DW) of plants transferred from pH 7 to pH 6 (○) or pH 9 (●). **(A) **Rb^+ ^content. **(B) **NO_3_^- ^content.

On the other hand, a broad range of K^+ ^channels have been shown to allow significant levels of NH_4_^+ ^to permeate [31], and at the same time Rb^+ ^is commonly used as a K^+ ^analogue in physiological studies [32], as its size and permeability characteristics are very similar to those of K^+^[33]. Thus we have used Rb^+ ^as a tracer for evaluating the effect of pH increase in cation uptake. The uptake rates of Rb^+ ^from the external RbNO_3 _source were similar at both pH levels throughout the experiment (Figure 5A). The anion (NO_3_^-^) absorption was lower under alkaline than acidic conditions (Figure 5B). In shoots, the internal NO_3_^- ^contents were similar in both external pHs (not shown). Therefore, all the effects observed in this study under NH_4_^+ ^nutrition and different pH conditions (Figures 3 and 4) can be just attributed to the ratio between NH_3 _and NH_4_^+^.

## Discussion

### Natural isotopic abundances of N in plants grown with NO_3_^- ^or NH_4_^+^

An important degree of fractionation, determined as the difference between the δ^15^N of the N source and that of the plant, was observed when plants were grown hydroponically with a known concentration of a single N form in a controlled environment (Figure 1). Thus, NO_3_^-^- fed plants tended to be enriched in the heavier N isotope in relation to the source, whereas NH_4_^+^-fed plants tended to be depleted (Figure 1).

The degree of fractionation in the reaction rates of the two N isotopes (^14^N and ^15^N) reflects both their mass differences and the force constants of the bonds they form. A significant isotope effect due to ionisation would therefore not be expected [34].

The positive δ^15^N values for NO_3_^-^-fed plants may be associated with N loss from the plant in the form of root efflux and exudates [6,7,35] or loss of NH_3 _through the stomata [36-39], which favours the lighter isotope [40]. The ratio between the root and shoot δ^15^N values may also depend on the partitioning of N metabolism between the roots and shoots. The isotopic effect for nitrate reductase enzyme is 1.015 (or higher, see [4] and references therein) and that associated with glutamine synthetase is 1.017 [41]; therefore, the resulting organic compounds (amino acids) would therefore be depleted of ^15^N in relation to the inorganic N pool. Thus, depending on the main site, shoots or roots, of N reduction and assimilation, the tissues would present distinct δ^15^N values. Since NO_3_^- ^and NH_4_^+ ^are not major constituents of the phloem, most of the N translocated into the plant in the organic form is likely to be depleted of ^15^N compared with N source. Because the main site of NO_3_^- ^reduction for each species is dependent on the N status of the plant, the relationship between the δ^15^N of roots and shoots may vary for the same plant species according to the external N availability and for the same external conditions according to plant species (Figure 1) and phenological stage. Thus, under NO_3_^- ^nutrition, there was no significant effect of the organ on the natural isotopic abundance of N (Table 2).

In contrast, the shoots of NH_4_^+^-fed plants were significantly enriched in ^15^N (Table 2) relative to the roots (see Additional file 3, tables S2 and S3). Among the various external factors, the source and concentration of N have an effect on stomatal NH_3 _emissions [36,37]. Thus, losses of NH_3 _from the stomata take place in NH_4_^+^-fed plants at high N concentrations [38,39]. This process will favour the lighter isotope emission and enrich the plant tissue (leaf specially) in ^15^N because the isotopic effect of NH_3 _(aq) exchange with NH_3 _(g) has been estimated to be 1.005. In other words, NH_3 _(g) is enriched in ^14^N by ~ 5 ‰ relative to NH_3 _(aq) [21]. In agreement with this reasoning, the nitrogen isotopic fractionation against ^15^N caused by volatilisation of NH_3 _has been shown in the aerial part of wheat plants [40]. Hence, in light of the N dynamics inside the plant, it is difficult to explain how the whole NH_4_^+^-fed plants can be depleted of the heavier N isotope.

### N Isotopic fractionation and NH_4_^+ ^toxicity mechanisms

Some studies have examined isotopic fractionation in plants grown with NH_4_^+ ^nutrition under LATS controlled conditions, and contrasting results were obtained. For instance, isotopic fractionation in NH_4_^+^-fed (4.6 mM) *Pinus sylvestris *ranged from 0.9 to 5.8 [42]. For *Oryza sativa *L., the fractionation was dependent on the external NH_4_^+ ^concentration, which ranged from -7.8 to -18 ‰ when the external NH_4_^+ ^concentrations ranged from 0.4 to 7.2 mM [7]. In agreement with this latter trend in rice, our results showed that the fractionation tended to increase with the N concentration for most of the plant species studied under NH_4_^+ ^nutrition (Figure 1, Table 2 and Additional file 3, tables S2 and S3). Hence, the organ δ^15^N values were closer to the source δ^15^N in low N availability conditions (at low N concentrations) for NH_4_^+^-fed plants [6] (Figure 1). Likewise, if the N concentration increases, the amount of substrate becomes unlimited and the isotope effect is observed [6] (Figure 1). However, the δ^15^N values from NO_3_^-^-fed plants were almost insensitive to the N concentration (Figure 1 and Table 2), which agrees with experiments in rice [7]. Thus, even if organic N compounds were lost, this phenomenon would not be sufficient to explain the plant depletion of ^15^N as the assimilatory enzymes discriminate against the heavier N isotope [4].

If we consider the mechanisms of NH_4_^+ ^toxicity, a recent study examined the causes of the primary root growth suppression by NH_4_^+ ^nutrition [43]. It demonstrated that the NH_4_^+^-mediated inhibition of primary root growth is mostly due to a repression of cell elongation rather than cell division inhibition. Moreover, these authors linked this phenomenon to two mechanisms of NH_4_^+ ^toxicity [44-46]. First, the futile plasma transmembrane cycle of NH_4_^+ ^uptake and efflux through cell roots, with the subsequent high energetic cost, might explain the different tolerances exhibited by different plant species when NH_4_^+ ^is supplied at high concentrations [44]. Hence, Li et al. [43] showed that NH_4_^+ ^efflux is induced by high NH_4_^+ ^concentrations in the *Arabidopsis *root elongation zone, which coincides with the inhibitory effect of NH_4_^+ ^on cell length and primary root elongation. They also associated the NH_4_^+^-induced efflux in the root elongation zone with the enzyme GDP-mannose pyrophosphorylase (GMPase). The implication of GMPase in the NH_4_^+ ^sensitivity of *Arabidopsis *roots represents the second (and last) mechanism of NH_4_^+ ^toxicity [45,46]. Therefore, Li et al. pointed out that GMPase regulates the process of root NH_4_^+ ^efflux, and showed that GMPase mutants had a higher net NH_4_^+ ^efflux (1.8 fold) in the root elongation zone relative to wild-type *Arabidopsis *plants [43].

In our study, we did not determine the net NH_4_^+ ^fluxes, but previous findings demonstrated that the root NH_4_^+^-induced efflux occurs in a broad range of plant species and are more or less significant depending on the NH_4_^+ ^sensitivity of the plant species [44]. So, the mechanism of NH_4_^+ ^ejection from the root cell, if it occurred, would significantly contribute towards the global ^15^N depletion of the NH_4_^+^-fed plants through a discriminatory mechanism against the lighter N isotope (i.e., favouring the ^15^N isotope). However, the fractionation mechanism against ^14^N is a thermodynamically unlikely event due to the differences in the physical and chemical properties of isotopic compounds. Thus, the heavier molecules have a lower diffusion velocity, and generally, the heavier molecules have higher binding energies [47].

Furthermore, the relative abundances of the stable isotopes in living organisms depend on the isotopic composition of their food sources and their internal fractionation processes [48]. Thus, taking into account the development of the relative abundance of the stable isotopes across the food web, internal fractionation generally leads to an enrichment of the heavier isotope in consumers relative to their diet [48]. The negative values for the natural isotopic fractionation observed in NH_4_^+^-fed plants must therefore be related to the chemical properties of the NH_4_^+ ^ion in solution and the NH_4_^+^/NH_3_-uptake mechanisms. When NH_4_^+ ^is applied as the only N source, the NH_4_^+ ^and NH_3 _forms are present in the nutrient solution. However, these molecular and ionic forms do not have exactly the same natural isotopic signatures because there is a 1.020 thermodynamic isotope effect between NH_3 _(aq) and NH_4_^+ ^(aq), such that NH_3 _(aq) is depleted of ^15^N by 20 ‰ relative to NH_4_^+ ^(aq) [20]. To interpret the negative values of the whole plant δ^15^N, we hypothesise that a portion of the N enters the root as NH_3_, which leads to the depletion of the heavier isotope in the plant.

### A proposal that relates N isotopic fractionation and NH_4_^+ ^toxicity mechanism

When the whole plant is considered and NH_4_^+ ^is the only available N source, the isotopic N signature of the plant would therefore be related to the amount of NH_3 _transported. Using the ratio between the biomass accumulations of NH_4_^+^- and NO_3_^-^-fed plants as an indicator of NH_4_^+ ^tolerance [22], we can relate NH_4_^+ ^tolerance to the root δ^15^N of NH_4_^+^-fed plants. Plants that were less tolerant to NH_4_^+ ^nutrition were the most depleted of the heavier isotope (Figure 2; Additional file 3, table S1), and presumably the uptake of NH_3 _was more important in those plants. According to our hypothesis, lettuce, spinach and tomato were the most sensitive to NH_4_^+ ^nutrition of the plant species studied (Figure 2 and Additional file 3 table S1). Moreover, the "plant sensitivity to NH_4_^+ ^nutrition" variable, expressed as the ratio of the biomasses of NH_4_^+^/NO_3_^-^-fed plants, can explain 69% of the root δ^15^N variation observed in the dataset (Figure 2). Hence, although the fraction of NH_3 _in solution at pH 6-7 is very small (approx. 0.07-0.6%), the transient alkalinisation of the cytosol reported after NH_3 _uptake can be attributed to rapid diffusion of NH_3 _across the plasma membrane and its subsequent protonation within the cytosol [49,50]. The increased NH_3 _concentration will therefore consume the established Δ μ_H+_, thereby contributing to a higher energetic cost to balance it. This may also be related to membrane depolarisation events observed after NH_4_^+ ^application in NH_4_^+^-tolerant plants or to the higher energetic burden reportedly required to maintain membrane potentials in NH_4_^+^-sensitive species [44].

In order to test the viability of our hypothesis, short-term experiments were performed using two plant species that showed different tolerance to NH_4_^+ ^nutrition at two pHs; a slightly acidic one pH (6.0), and an alkaline pH (9.0) which favoured the neutral form (NH_3_). Spinach (sensitive; Figure 2) and pea (tolerant; Figure 2) receiving ^15^NH_4_^+ ^as the only N source showed that 2 h was sufficient to demonstrate that N uptake was faster in plants transferred from pH 6-7 to pH 9 than in those transferred from pH 6-7 to pH 6 (Figure 3B and 3C). The differences shown in shoot ^15^N contents between pHs and species (Figure 3A and 3C) suggest interesting dissimilarities in uptake and transport systems, linked to the degree of sensitivity/tolerance of these species to NH_4_^+^. This finding may be related to the different distribution of incorporated NH_4_^+ ^reported in both species (shoot in spinach and root in pea plants) [51]. In this work it is proposed that differences in the site of NH_4_^+ ^assimilation is linked to NH_4_^+ ^tolerance. On the other hand, taking into consideration the N absorbed by the plants and the dissociation constant of the ionic form, most of the difference in N uptake at pH 6 and pH 9 is likely related to a higher proportion of NH_3 _under alkaline conditions (Figure 4B and 4D). These observations are consistent with the hypothesis that the NH_3 _form is involved in the uptake of reduced N by the cell in the LATS activity range.

Physiological studies have indicated that transport of NH_3 _across membranes occurs and may become significant at high NH_4_^+ ^concentrations or at high pHs [16]. Indeed, NH_3 _transport has been described as a function of the HATS in *Escherichia coli *[52,53]. The first hints of protein involvement in plant NH_3 _transport came from nodules of legume rhizobia symbiosis and restoration of NH_3 _transport in yeast mutants complemented with three aquaporins from wheat roots. This complementation was found to be pH-dependent, with progressively better growth being observed at increasing pH, and was thus indicative of transport of neutral NH_3 _rather than charged NH_4_^+^[54]. Recently, the transport of NH_3_, rather than NH_4_^+^, by the AtAMT2 transporter was also shown [14,15]. Furthermore, the incubation of an illuminated suspension of mesophyll cell protoplasts from *Digitaria sanguinalis*, which had been preloaded with a pH-specific fluorescent probe, with 20 mM of NH_4_Cl showed rapid alkalinisation of the cytosolic pH [55], which may be explained on the basis of NH_3 _uptake. Further examples of transient alkalinisation of the cytosol have been reported in root hair cells of rice and maize after the addition of 2 mM NH_4_^+ ^to a previously N-free bathing solution [50], which indicates that NH_3 _permeates cells [50,55]. This process will contribute to consumption of the established Δ μH^+ ^and agrees with the hypothesis that the toxic effect of NH_3 _is associated with intracellular pH changes [44]. All of these studies together demonstrate that NH_4_^+ ^may permeate cells in its neutral form (NH_3_) and therefore tends to increase cytosolic pH.

The level of GMPase activity has been proposed to be a key factor in the regulation of *Arabidopsis *sensitivity to NH_4_^+^[45]. Interestingly, these authors showed that GMPase activity is seemingly regulated by pH. Using *in vitro *experiments with recombinant wild-type and GMPase mutant proteins, GMPase activity was decreased by alkaline pH. In plants cultured on NO_3_^-^, a considerable decrease in GMPase activity was observed with increasing pHs from 5.7 to 6.7 of the plant growth medium. Moreover, plants grown in the presence of NH_4_^+ ^showed lower GMPase activities relative to that shown by NO_3_^-^-fed plants at the same external pH [45]. This could indicate that the transient cytosolic alkalinisation previously reported in NH_4_^+ ^uptake (reviewed in [56]) may trigger the decrease of GMPase activity stimulated by NH_4_^+ ^provision [45]. In fact, Qin et al. have hypothesised that this cytosolic alkalinisation may play a role in the inhibition of GMPase activity by NH_4_^+^[45].

Thus, in view of our results and these previous findings, we propose the existence of a mechanism that recruited the NH_4_^+ ^in the molecular form (NH_3_) under LATS conditions, which would cause in parallel depletion in the heavier N isotope, as well as an alkalinisation of cytosol in root cells. It would trigger a decrease in GMPase activity and the subsequent downstream molecular events, i.e., deficiencies in protein N-glycosylation, the unfolded protein response and cell death in the roots [45], which are important for the inhibition of *Arabidopsis *growth by NH_4_^+ ^application [45]. Moreover, reductions in cellulose biosynthesis, cell wall stability and cell viability shown in a null mutant of GMPase (*cty1-2*) are the result of an N-glycosylation deficiency [57]. The disturbance of cell wall biosynthesis caused by the decreased GMPase activity under NH_4_^+ ^nutrition and the subsequent protein N-glycosylation deficiency [45] has been related to the NH_4_^+ ^flux [43]. Our proposal, therefore, is compatible with the two related NH_4_^+^-toxicity mechanisms [43] proposed by Britto et al. [44] and Qin et al. [45].

On the other hand, several reports have suggested that K^+ ^channels are an important component of the LATS for NH_4_^+^[58]. It has been shown that NH_4_^+ ^produces similar, but weaker, currents compared to K^+ ^in intact root cells or in protoplasts ([10] and references therein) and that a single amino acid substitution in a K^+ ^channel can dramatically increase NH_4_^+ ^permeability [59]. Indeed, a broad range of K^+ ^channels have been shown to be permeable to NH_4_^+^[8,60], and most allow significant levels of NH_4_^+ ^to permeate [31]. Alternatively, it might be expected that some channels and transporters poorly distinguish between K^+ ^and NH_4_^+^. In fact, it has been shown that the futile NH_4_^+ ^cycling, which was shown in NH_4_^+^-sensitive plants under NH_4_^+ ^nutrition [44], is alleviated by elevated K^+ ^levels and that low-affinity NH_4_^+ ^transport is mediated by two components, one of which is K^+ ^sensitive and the other is K^+ ^independent [31]. As NH_4_^+ ^transport through K^+ ^channels would be in the ionic form, no ^15^N fractionation is expected to be associated with it.

## Conclusions

Based on the results presented herein, we show that plants fed with NH_4_^+ ^as the sole source of N are depleted of ^15^N in a concentration-dependent manner. We have observed a relationship between ^14^N/^15^N fractionation and the sensitivity of plants to NH_4_^+ ^nutrition. We show that the most sensitive plants have the most negative δ^15^N values. Moreover, our data of ^15^N uptake at pH 6.0 and 9.0 together with other data found in the literature indicate that part of N uptake by the plant may occurs as NH_3_. Accordingly, current data has suggested that the LATS for NH_4_^+ ^has at least two components. One component is involved in the transport of NH_3 _and would therefore indirectly discriminate against the heaviest N stable isotope due to the balance between ionic and molecular forms in the nutrient solution. This transport mechanism could correspond to the K^+^-independent component of NH_4_^+ ^transport suggested previously [31]. The second component would be an NH_4_^+^-specific transport system, which interferes with K^+ ^transport and does not discriminate against ^15^N. We propose that the negative values of δ^15^N observed in hydroponically grown plants are related to this NH_3 _uptake, which imprints a permanent N signature (δ^15^N) under steady-state external N conditions and contributes to the current understanding of the origin of NH_4_^+ ^toxicity.

## Authors' contributions

IA participated in experimental design and its coordination, carried out the short-term ^15^N labelling experiments and participated in isotopic signature experiments, analysed the data, performed the statistical analysis and wrote the paper. CC conceived of the study, carried out the isotopic signature experiments, analysed the data and wrote the manuscript. JFM conceived of the study and wrote the manuscript. MBG-M participated in the isotopic signature experiments and helped to draft the paper. CG-O performed the statistical analysis. CG-M carried out the isotopic signature experiments. MAM-L participated in isotopic signature experiments and helped to draft the paper. PMA-T conceived of the study, designed and coordinated the experiments, conducted the short-term ^15^N labelling and the isotopic signature experiments and helped to write the manuscript. All authors have read and approved the final manuscript.

## Supplementary Material

Additional file 1 **Control measures of external pH in all short-term experiments**. Initial and final pH values of the external solutions at pH 6 (panels A, C and E) and 9 (panels B, D and F). Click here for file

Additional file 2 **Calculations appendix**. The calculations used to achieve these results have been added to the manuscript to clarify the discussion and conclusions of this work. **A) **Calculations for obtaining the ^15^N content as μmol ^15^N·100 g^-1 ^DW from the δ^15^N (‰) and total N content (% N). **B) **The ^15^N contents from the external NH_4_^+ ^and NH_3 _were calculated using the Henderson-Hasselbalch equation to take into account the external pH conditions. Click here for file

Additional file 3 **Natural isotopic signature data**. Tables with plant biomass ratios of plants fed with NH_4_^+^/NO_3_^- ^as the sole N source and δ^15^N values in shoots and roots of plants fed with NH_4_^+ ^or NO_3_^- ^as the sole N source. Click here for file
