# Supplementary figures and images for: Depletion of the heaviest stable N isotope is associated with NH4+/NH3 toxicity in NH4+-fed plants
Source: BMC Plant Biol. 2011 May 16;11:83. doi: 10.1186/1471-2229-11-83 (PMC3224212; doi:10.1186/1471-2229-11-83)

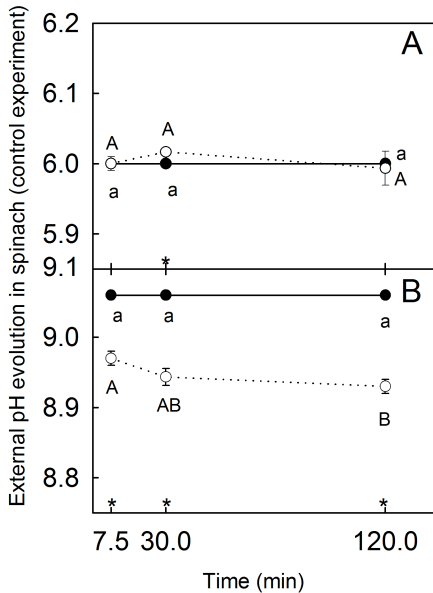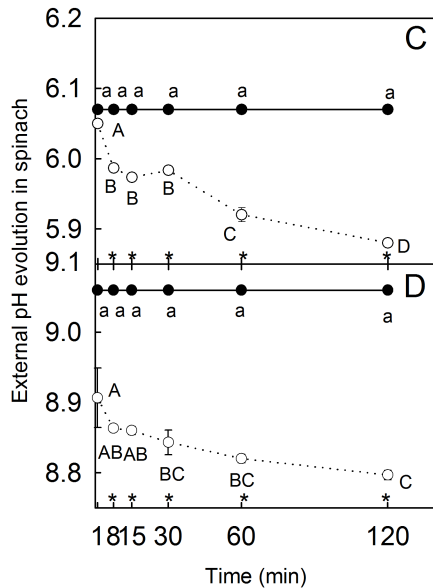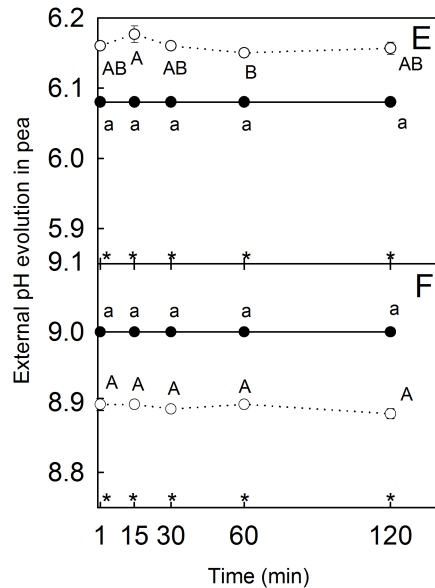

—●— Initial pH ···○··· Final pH

Supplement: Additional file 1 — Control measures of external pH in all short-term experiments. Initial and final pH values of the external solutions at pH 6 (panels A, C and E) and 9 (panels B, D and F). [file 1471-2229-11-83-S1.PDF]
