# Supplementary material for: Depletion of the heaviest stable N isotope is associated with NH4+/NH3 toxicity in NH4+-fed plants
Source: BMC Plant Biol. 2011 May 16;11:83. doi: 10.1186/1471-2229-11-83 (PMC3224212; doi:10.1186/1471-2229-11-83)
Supplement: Additional file 2 — Calculations appendix. The calculations used to achieve these results have been added to the manuscript to clarify the discussion and conclusions of this work. A) Calculations for obtaining the 15N content as μmol 15N·100 g-1 DW from the δ15N (‰) and total N content (% N). B) The 15N contents from the external NH4+ and NH3 were calculated using the Henderson-Hasselbalch equation to take into account the external pH conditions. [file 1471-2229-11-83-S2.PDF]

Additional file 2. Calculations

A) For obtaining ^{15}N content as μmol $^{15}N \cdot 100\ g^{-1}$ DW from $\delta^{15}N$ (‰) and total N content (% N) the following formula was used,

$$\delta\,(\text{‰}) = \left[\frac{R_s}{R_{std}} - 1\right] \times 1000 \quad \textbf{(Eqn 1)}$$

Where,

$$R_s = {}^{15}N_{sample} \big/ {}^{14}N_{sample}$$

$$R_{std} = \frac{{}^{15}N_{std}}{{}^{14}N_{std}} = \frac{1}{272} = 0.0036765\ (\textit{atmospheric relative amount of}\ {}^{15}N)$$

On the other hand, making the following change:

$$a = \%\ {}^{15}N\left(\frac{\textit{molecules of}\ {}^{15}N}{100\ \textit{molecules of total}\ N}\right)\ \textit{then},\ \%{}^{14}N = 100 - a \quad \textbf{(Eqn 2)}$$

Combinating **(Eqn 1)** and **(Eqn 2)**,

$$\delta\,(\text{‰}) = \left[\frac{\left(\frac{a}{100-a}\right)}{0.003676} - 1\right] \times 1000$$

And finally, isolating the variable,

$$a = \frac{0.3676\ \delta^{15}N + 367.6}{0.003676\ \delta^{15}N + 1003.676}$$

For obtaining the % of ^{15}N as μmol $^{15}N \cdot 100\ g^{-1}$ DW from $\delta^{15}N$ (‰), it is also necessary the total N content (% N as mol total N/100g DW),

$$\%\ {}^{15}N\left(\frac{molecules\ {}^{15}N}{100 molecules\ N_{total}}\right)\times\%\ N\left(\frac{molecules\ N_{total}}{100\ g\ DW}\right)=\frac{molecules\ {}^{15}N}{100\ g\ DW}$$

B) The ^{15}N contents from external NH_4^+ and NH_3 were calculated using the Henderson-Hasselbalch equation taking into account external pH conditions as follows,

$$NH_4^+ + H_2O \xleftrightarrow{pKa\ 9.25} NH_3 + H_3O^+ \quad \textbf{(Eqn 3)}$$

$$pH = pKa + \log\left(\frac{[NH_3]}{[NH_4^+]}\right) \quad \textbf{(Eqn 4)}$$

$$\begin{aligned}
&At\ pH\ 6,\\
&6.08 = 9.25 + \log\left(\frac{NH_3}{NH_4^+}\right)\\
&And\ \ final\ equation\ is:\\
&[NH_3] = 0.000676083[NH_4^+]\\
&For\ each\ NH_4^+\ molecule,\ there\ are\ 0.000676083\ of\ NH_3,\\
&so\ each\ 100\ molecules\ of\ NH_4^+,\ there\ are\ 0.676083\ of\ NH_3
\end{aligned}$$

On the other hand,

$$100 = [NH_3] + [NH_4^+] \Rightarrow [NH_3] = 100 - [NH_4^+] \quad \textbf{(Eqn 5)}$$

Then the percentage of molecules in each form (neutral or acid), is calculated as combination of **(Eqn 4)** and (**Eqn 5**),

$$[NH_4^+] = \frac{100}{1.000676083} = 99.93244\%$$

$$(99.93244\, molecules\ of\ NH_4^+\ for\ 100\ molecules\ of\ total\ N\ in\ an\ ammoniacal\ solution)$$

$$[NH_3] = 100 - 99.93244 = 0.06756\%$$

$$(0.06756\ molecules\ of\ NH_3\ for\ 100\ molecules\ of\ total\ N\ in\ an\ ammoniacal\ solution)$$

For obtaining the % of $^{15}NH_3$ as µmol $^{15}NH_3 \cdot 100\ g^{-1}$ DW from $\delta^{15}N$ (‰) and total N content

(% N),

$$\%\,^{15}NH_3\left(\frac{\mu mol\ ^{15}NH_3}{100 g^{-1} DW}\right) = \%^{15}N\left(\frac{mol\ ^{15}Ntotal}{100 mol\ Ntotal}\right) \times \%N\left(\frac{mol\ Ntotal}{100\ g\ DW}\right) \times \%^{15}NH_3\left(\frac{mol\ ^{15}NH_3}{100 mol\ ^{15}Ntotal}\right) \times 10^6\left(\frac{\mu mol^{15}NH_3}{mol^{15}NH_3}\right)$$

(Eqn 6)
