# Supplementary material for: Depletion of the heaviest stable N isotope is associated with NH4+/NH3 toxicity in NH4+-fed plants
Source: BMC Plant Biol. 2011 May 16;11:83. doi: 10.1186/1471-2229-11-83 (PMC3224212; doi:10.1186/1471-2229-11-83)
Supplement: Additional file 3 — Natural isotopic signature data. Tables with plant biomass ratios of plants fed with NH4+/NO3- as the sole N source and δ15N values in shoots and roots of plants fed with NH4+ or NO3- as the sole N source. [file 1471-2229-11-83-S3.PDF]

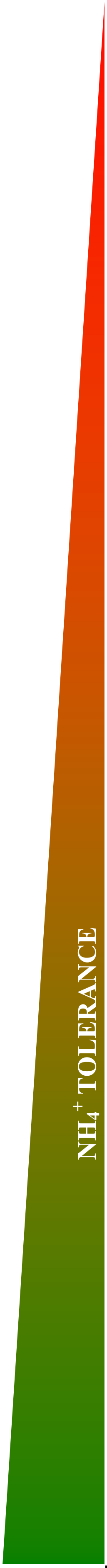

Species	[N] (mM)	Root δ ^{15}N (‰)	Plant biomass ratio
Lactuca sativa L. cv. Marine	0.5	-6.81 ± 0.33	0.78 ± 0.004
	1.5	-11.57 ± 0.13	0.54 ± 0.014
	3	-14.04 ± 0.30	0.38 ± 0.009
Spinacia oleracea L. cv. Gigante de invierno	1.5	-9.47 ± 0.06	0.23 ± 0.037
	3	-8.89 ± 0.01	0.07 ± 0.003
Spinacia oleracea L. cv. Spinner	0.5	-4.88 ± 0.32	1.16 ± 0.350
	1.5	-8.18 ± 0.08	0.72 ± 0.052
	3	-9.556 ± 0.10	0.56 ± 0.016
	6	-12.40 ± 0.44	0.28 ± 0.004
Solanum lycopersicum L. cv. Trust	0.5	-3.29 ± 0.10	1.17 ± 0.027
	1.5	-4.33 ± 0.12	1.12 ± 0.019
	3	-7.96 ± 0.25	0.66 ± 0.055
	6	-9.11 ± 0.49	0.55 ± 0.021
Lolium perenne L. cv. Herbus	0.5	-2.70 ± 0.68	1.02 ± 0.157
	2.5	-7.34 ± 1.94	0.76 ± 0.149
	5	-7.90 ± 1.20	0.83 ± 0.057
Pisum sativum L. cv. Snap-pea	2.5	-3.95 ± 0.05	1.06 ± 0.128
	5	-9.25 ± 0.05	0.92 ± 0.040
Pisum sativum L. cv. Rondo	1.5	-0.21 ± 0.17	1.21 ± 0.036
	3	-3.92 ± 0.11	0.88 ± 0.030
Pisum sativum L. cv. Eclipse	0.5	-3.59 ± 0.23	1.41 ± 0.047
	1.5	-3.71 ± 0.09	1.20 ± 0.050
	3	-5.089 ± 0.18	1.14 ± 0.023
	6	-6.16 ± 0.06	0.96 ± 0.018
Lupinus albus L. cv. Albus	0.5	-1.01 ± 0.012	1.25 ± 0.066
	1.5	-1.06 ± 0.01	1.24 ± 0.135
	3	-2.45 ± 0.02	1.17 ± 0.022
	6	-3.17 ± 0.01	1.17 ± 0.025
Trifolium repens L. cv. Huia	5	-5.87 ± 1.68	1.06 ± 0.190
Ceratonia siliqua sp.	1.5	-4.71 ± 0.12	2.53 ± 0.720
	3	-3.89 ± 0.23	2.62 ± 0.570

Additional file 3. Table S1

Informative heading:

Additional file 3. Table S1. Root isotopic signature ($\delta^{15}N$, ‰) and plant biomass ratio of plants fed with NH_4^+/NO_3^- as the sole N source.

Footnote:

Plant species (*Lactuca sativa* L., *Spinacia oleracea* L., *Solanum lycopersicum* L., *Lolium perenne* L., *Pisum sativum* L., *Lupinus albus* L., *Trifolium repens* L. and *Ceratonia siliqua* sp.) were cultured hydroponically with different N concentrations. Data represent average values ± SE (at least n = 3, depending on species; see Methods). This data set is correlated in Figure 2.

Species	[N] (mM)	Shoot δ ^{15}N (‰) NH_4^+-fed plants	Shoot δ ^{15}N (‰) NO_3^--fed plants
Lactuca sativa L. cv. Marine	0.5	-6.02± 0.45	3.15 ± 0.32
	1.5	-7.38 ± 0.65	5.02 ± 0.48
	3	-7.20 ± 0.65	3.12 ± 0.41
Spinacia oleracea L. cv. Gigante de invierno	1.5	-5.56 ± 0.15	4.93 ± 0.23
	3	-5.61 ± 0.04	4.80 ± 0.27
Spinacia oleracea L. cv. Spinner	0.5	-4.01 ± 0.12	3.11 ± 0.32
	1.5	-5.12 ± 0.54	4.93 ± 0.32
	3	-5.62 ± 0.45	4.89 ± 0.67
	6	-9.36 ± 0.54	4.89 ± 0.66
Solanum lycopersicon L. cv. Trust	0.5	-3.00 ± 0.06	5.12 ± 0.37
	1.5	-3.45 ± 0.12	7.32 ± 0.39
	3	-5.45 ± 0.76	8.01 ± 1.01
	6	-8.15 ± 0.76	6.15 ± 0.67
Lolium perenne L. cv. Herbus	0.5	-4.16 ± 0.65	12.30 ± 0.00
	2.5	-10.39 ± 1.71	11.10 ± 0.00
	5	-12.12 ± 1.38	11.00 ± 0.10
Pisum sativum L. cv. Snap-pea	0.5	-0.85 ± 0.05	-
	2.5	-5.75 ± 0.15	-
	5	-10.00 ± 0.00	-
Pisum sativum L. cv. Rondo	1.5	-0.27 ± 0.02	10.75 ± 0.14
	3	-4.59 ± 0.08	12.00 ± 0.12
Pisum sativum L. cv. Eclipse	0.5	-3.32 ± 0.32	10.01 ± 1.53
	1.5	-0.21 ± 0.04	10.65 ± 0.43
	3	-3.89 ± 0.09	11.77 ± 1.66
	6	-3.32 ± 0.28	12.06 ± 1.43
Lupinus albus L. cv. Albus	0.5	-0.10 ± 0.01	-0.12 ± 0.01
	1.5	-0.30 ± 0.01	12.90 ± 1.23
	3	-3.12 ± 0.23	13.10 ± 1.13
	6	-3.15 ± 0.24	13.78 ± 1.66
Trifolium repens L. cv. Huia	0.5	-1.90 ± 1.02	11.12 ± 0.76
	2.5	-5.95 ± 1.64	11.51 ± 0.87
	5	-6.76 ± 0.82	11.15 ± 0.67
Ceratonia siliqua sp.	1.5	-2.76 ± 0.23	2.54 ± 0.14
	3	-2.99 ± 0.15	3.15 ± 0.11
Acacia neura sp.	1.5	-1.18 ± 0.03	2.88 ± 0.23
	3	-0.92 ± 0.11	2.84 ± 0.18

Additional file 3. Table S2

Informative heading:

Additional file 3. Table S2. Natural isotopic abundance ($\delta^{15}N$, ‰) in shoots of plants fed with NH_4^+ or NO_3^- as the sole N source.

Footnote:

Plant species (*Lactuca sativa* L., *Spinacia oleracea* L., *Solanum lycopersicum* L., *Lolium perenne* L., *Pisum sativum* L., *Lupinus albus* L., *Trifolium repens* L., *Ceratonia siliqua* sp. and *Acacia aneura* sp. were cultured hydroponically with different N concentrations.
Data represent average values ± SE (at least n = 3, depending on species; see Methods). This data set is displayed in Figure 1A.

Species	[N] (mM)	Root δ ^{15}N (‰) NH_4^+-fed plants	Root δ ^{15}N (‰) NO_3^--fed plants
Lactuca sativa L. cv. Marine	0.5	-6.78 ± 0.53	3.14 ± 0.55
	1.5	-12.05 ± 1.54	4.81 ± 0.38
	3	-14.54 ± 2.35	2.99 ± 0.34
Spinacia oleracea L. cv. Gigante de invierno	1.5	-9.47 ± 0.11	3.71 ± 0.15
	3	-8.89 ± 0.02	3.48 ± 0.23
Spinacia oleracea L. cv. Spinner	0.5	-4.8 ± 0.43	3.28 ± 0.51
	1.5	-9.77 ± 1.13	3.26 ± 0.12
	3	-8.12 ± 1.01	3.55 ± 0.33
	6	-12.39 ± 1.13	4.56 ± 0.55
Solanum lycopersicum L. cv. Trust	0.5	-3.30 ± 0.12	5.33 ± 0.71
	1.5	-6.54 ± 0.55	7.51 ± 1.01
	3	-6.12 ± 0.82	6.54 ± 0.55
	6	-9.10 ± 1.12	6.29 ± 1.08
Lolium perenne L. cv .Herbus	0.5	-2.70 ± 0.68	10.71 ± 0.29
	2.5	-7.34 ± 1.93	10.60 ± 0.00
	5	-10.38 ± 1.20	9.62 ± 0.12
Pisum sativum L. cv. Snap-pea	0.5	-1.75 ± 0.05	-
	2.5	-3.95 ± 0.05	-
	5	-9.25 ± 0.05	-
Pisum sativum L. cv. Rondo	1.5	-0.21 ± 0.17	9.23 ± 0.16
	3	-3.92 ± 0.11	10.02 ± 0.10
Pisum sativum L. cv. Eclipse	0.5	-3.54 ± 0.19	8.51 ± 0.67
	1.5	-2.04 ± 0.33	9.23 ± 1.12
	3	-3.45 ± 0.55	10.00 ± 1.23
	6	-7.18 ± 0.87	11.32 ± 1.21
Lupinus albus L. cv. Albus	0.5	-0.12 ± 0.04	5.32 ± 0.44
	1.5	0.03 ± 0.04	8.12 ± 0.56
	3	-3.32 ± 0.06	11.24 ± 1.08
	6	-4.16 ± 0.56	13.12 ± 1.28
Trifolium repens L. cv. Huia	0.5	-0.66 ± 0.02	10.65 ± 0.83
	2.5	-0.64 ± 0.42	8.34 ± 0.67
	5	-5.87 ± 1.68	4.97 ± 0.00
Ceratonia siliqua sp.	1.5	-4.82 ± 0.35	2.75 ± 0.34
	3	-3.79 ± 0.25	3.79 ± 0.45
Acacia neura sp.	1.5	-1.09 ± 0.23	2.77 ± 0.28
	3	-0.88 ± 0.11	2.76 ± 0.14

Additional file 3. Table S3

Informative heading:

Additional file 3. Table S3. Natural isotopic abundance ($\delta^{15}N$, ‰) in roots of plants fed with NH_4^+ or NO_3^- as the sole N source.

Footnote:

Plant species (*Lactuca sativa* L., *Spinacia oleracea* L., *Solanum lycopersicum* L., *Lolium perenne* L., *Pisum sativum* L., *Lupinus albus* L., *Trifolium repens* L., *Ceratonia siliqua* sp. and *Acacia neura* sp.) were cultured hydroponically with different N concentrations.
Data represent average values ± SE (at least n = 3, depending on species; see Methods). This data set is displayed in Figure 1B.
